# Controlling and synchronizing a fractional-order chaotic system using stability theory of a time-varying fractional-order system

**DOI:** 10.1371/journal.pone.0194112

**Published:** 2018-03-30

**Authors:** Yu Huang, Dongfeng Wang, Jinying Zhang, Feng Guo

**Affiliations:** 1 Department of Automation, North China Electric Power University, Baoding, China; 2 Department of Automation, North China Electric Power University, Baoding, China; 3 Shenhua Guohua Electric Power Research Institute Corporation, Beijing, China; 4 Department of Cognitive Science, School of Information Science and Engineering, Xiamen University, Xiamen, China; Lanzhou University of Technology, CHINA

## Abstract

Control and synchronization of fractional-order chaotic systems have attracted wide attention due to their numerous potential applications. To get suitable control method and parameters for fractional-order chaotic systems, the stability analysis of time-varying fractional-order systems should be discussed in the first place. Therefore, this paper analyzes the stability of the time-varying fractional-order systems and presents a stability theorem for the system with the order 0<*α*<1. This theorem is a sufficient condition which can discriminate the stability of time-varying systems conveniently. Feedback controllers are designed for control and synchronization of the fractional-order Lü chaotic system. The simulation results demonstrate the effectiveness of the proposed theorem.

## 1. Introduction

Fractional-order calculus which extends the descriptive abilities of integer-order calculus can be traced to the work of Leibniz and Hospital in 1695. The integer-order calculus depends only on the local characteristics of a function’s, but fractional-order calculus accumulates all information of the function in a certain time, which is also called memory property. Mathematical models based on fractional-order calculus can describe the dynamic behavior of an actual system accurately in many areas, thereby it is necessary to facilitate the improvement of its design and control stability for fractional-order dynamic systems [1]. Recently, fractional-order chaotic control and synchronization have attracted increasing attention. In [2], Razminia A *et al*. synchronized a unidirectional coupling structure for the two fractional order chaotic systems using a sliding mode control methodology. In [3], Wu GC *et al*. presented a nonlinear coupling method to study the master-slave synchronization for the fractional differential equation. In [4], Golmankhaneh AK *et al*. have presented the chaos synchronization of two identical and nonidentical fractional orders of a new chaotic system by using active control. In [5], Jajarmi A *et al*. used a linear state feedback controller together with an active control technique in order to control a hyperchaotic financial system. In [6], a Lyapunov approach is adopted for deriving the parameter adaptation laws and proving the stability of the generalized projective synchronization (GPS) of two incommensurate fractional-order chaotic closed-loop systems. A linear feedback controller is proposed to achieve synchronisation of a fractional-order system with uncertainties and disturbance and guarantees the bounded state error for any bounded interference infinite time [7]. In [8], a simple but practical method to synchronize almost all familiar fractional-order chaotic systems which are including the commensurate system and incommensurate case, autonomous system, and the nonautonomous case has been put forward, and sufficient conditions are derived to guarantee synchronization of these systems. In [9], Shao SY *et al*. studies the fractional-order disturbance observer (FODO)-based adaptive sliding mode synchronization control for a class of fractional-order chaotic systems with unknown bounded disturbances. In [10], Soukkou A *et al*. proposed a fractional-order prediction-based feedback control scheme (Fo-PbFC) to stabilize the unstable equilibrium points and to synchronize the fractional-order chaotic systems (FoCS). In [11], Nourian *et al*. estimated the unknown coefficients of the system and demonstrated the stabilization of the synchronizer system by using the adaptive rule and a proper Lyapunov candidate function. In [12], Maheri *et al*. put forward a robust adaptive nonlinear feedback controller scheme to realize the synchronization of two different fractional-order chaotic systems in the condition of fully unknown parameters, external disturbance and uncertainties. In [13], Zhou *et al*. designed an adaptive controller to synchronize two entirely different fractional-order chaotic systems with uncertain parameters. Combining with appropriate parameter estimation laws. In [14], Yang proposed a single-state proportional feedback method to synchronize two identical generalized Lorenz systems. Used Lyapunov stability theory and a fractional-order differential inequality. In [15], Zhang *et al*. developed a modified adaptive control scheme and adaptive parameter laws to robustly synchronize coupled with fractional-order chaotic systems without certain parameters and perturbations. In [16], Xiang *et al*. investigated a robust synchronization for a class of systems with external disturbances.

In addition, many scholars have made great contributions in the field of the control and stability of time-varying fractional order systems. In [17], Aguila-Camacho N *et al*. put forward a new lemma for the Caputo fractional derivatives which has been proved to be useful in order to find the fractional-order extension of Lyapunov functions and can be used to demonstrate the stability of many fractional order systems including nonlinear and time-varying. In [18], Bao HB *et al*. put forward sufficient conditions which ensure the drive–response systems to achieve adaptive synchronization of fractional-order memristor-based neural networks with time-varying delay. In [19], the authors dealt with the fractional-order neural networks with impulsive effects and time-varying delay, and established several sufficient conditions guaranteeing the global Mittag–Leffler stability of the equilibrium point of the neural networks.

However, the most basic control and synchronization problem of chaotic systems are that of stability. Stability is a precondition for normal operation of systems and the main factor of system designs. A Lyapunov direct method is a core issue in integer-order stability theory, which is also a basic stability theorem for control systems.

It has been proven that the Lyapunov direct method is a relatively complete theoretical for integer-order systems both in theoretical study and practical application. As the transfer function of fractional-order systems is usually not a rational function of complex variable *s*, the stability analysis of fractional-order systems is far more complicated than that of integer-order systems. Many scholars have carried out extensive research on time-invariant fractional-order systems and made considerable achievements. For fractional-order LTI systems, in [20], Semary *et al*. discussed their physical and non-physical transfer functions, stability, poles, time domain, frequency domain, their relationships for different fractional-order differential equations and other basic concepts. In [21], Wang *et al*. used the argument principle of complex analysis to deduce two stability criteria for linear time-invariant fractional-order systems, which can determine system stability without utilizing characteristic roots. They also used Laplace transform and residue theorem to discuss the internal and external stability conditions of a linear time-invariant fractional-order system [22]. Pakzad put forward a practical analytical model to discuss the stability robustness of a class of linear time-invariant fractional-order systems with single and multiple commensurate delays of retarded type, against delay uncertainties [23].

All the above stability analyses are for time-invariant fractional-order systems. However, the above results are not widely used due to various reasons. For example, the eigenvalue criterion cannot be applied in time-varying fractional-order systems [24]. Therefore, this paper analyzes the stability of the time-varying fractional-order systems and presents a stability theorem for the system with the order 0<*α*<1. This theorem is a sufficient condition which can discriminate the stability of time-varying systems conveniently. Feedback controllers are designed for control and synchronization of the fractional-order Lü chaotic system.

The rest of the paper is organized as follows. Section 2 analyzes the development status and the stability of fractional-order systems. Section 3 presents a stability theorem for these systems with the order 0<*α*<1. Feedback controllers for fractional-order Lü chaotic system’s control and synchronization are designed on the basis of previous stability theorem in Section 4. Finally, the conclusion is drawn according to the present study in Section 5.

## 2. Development status of fractional-order system and stability

### 2.1 Definition of fractional-order calculus

Nowadays, many different definitions of fractional-order calculation were presented, in [25]. The most common definition, with αϵ(0,1), is shown as Eq 1 and was proposed by M. Caputo in 1967. Eq 1 is important for integral transformation because the initial value expressions generated in integral transformation are all in the form of integer-order derivatives, which can be effectively applied in practice.

$${}_{t_{0}}I_{t}^{\alpha }x(t)=\frac{1}{\Gamma (\alpha )}\int_{t_{0}}^{t}\frac{x(\tau )}{{\left(t-\tau \right)}^{1-\alpha }}d\tau ,$$ (1)

Where *x*(*t*) is a function with an arbitrary integer order; the fractional order meets 0<*α*<1; _t_0__*I*_*t*_^*α*^ is a fractional-order integral with order _*α*_ of function *x*(*t*) between [*t*_*0*_,*t*]; Γ (·); denotes the gamma function.

**Definition 1** For any real number *q*, denotes the integer part of *q*, that is to say,[q]is the largest integer no more than *q*. ${}_{t_{0}}D_{t}^{\alpha }$is a Caputo fractional differential operator. Thus, the differential of *x*(*t*) with fractional-order *q* is

$${}_{t_{0}}D_{t}^{q}x(t)=\frac{1}{\Gamma (\left\lfloor q\right\rfloor +1-q)}\int_{t_{0}}^{t}\frac{x^{\left(\left\lfloor q\right\rfloor +1\right)}(\tau )}{{\left(t-\tau \right)}^{q-\left\lfloor q\right\rfloor }}d\tau$$ (2)

### 2.2 Development of stability analysis of fractional-order system

**Theorem 1** When 0<α<1, x∊R^n^, *A*∊R^nxn^, the fractional-order system _t_0__*D*^α^_t_x(t) = *A*x(t), t≥t_0_ is asymptotically stable if and only if all the characteristic values of matrix *A* satisfy |arg(eig(*A*))|>απ/2. Furthermore, the system is stable if and only if all the characteristic values of matrix *A* satisfy |arg(eig(*A*))|≥απ/2, which can be found in [21].

Theorem 1 is the existing stability criterion of a linear time-invariant fractional-order system with 0<α<1. This theorem is suitable only for a linear time-invariant fractional-order system, but it is often misused [26]. For time-invariant fractional-order nonlinear systems, if all the eigenvalues of the Jacobi matrix at equilibrium are stable, then the equilibrium is called stable equilibrium point. However, Theorem 1 is not suitable for time-varying fractional-order systems.

Considering the time-varying fractional-order system with order 0<α<1 and initial value *x*(*t*_0_), the following is obtained:

$${}_{t_{0}}D_{t}^{\alpha }x(t)=f(t,x)$$ (3)

Where α∊(0,1), *f*:[t_0,_∞]xΩ→R^n^ is piecewise continuous and meets the local Lipschitz condition (Ω∊*R*^n^is a domain that contains *x* = 0).

**Definition 2** A continuous function *β*: [0,*t*)→[0,∞) is said to belong to class-k if it is strictly increasing and *β*(0) = 0.

**Definition 3** If and only if $f(t,x_{e})={}_{t_{0}}D_{t}^{\alpha }x_{e}$, then constant *x*_*e*_ is the equilibrium point of the Caputo-defined fractional-order dynamic system (3). Without loss of generality, we assume *x*_*e*_ *=* 0.

**Theorem 2 [25]** Let *x*_*e*_ *=* 0 be an equilibrium point of the fractional-order system (3). Assume that Lyapunov function *V*(*t*,*x*(*t*)) and class-k functions *β*_*i*_(*i* = 1, 2, 3) exist, which satisfy

$$\beta_{\text{1}}(\left\|x\right\|)\leq V(t,x)\leq \beta_{\text{2}}(\left\|x\right\|),$$ (4)

$${}_{0}D_{t}^{\gamma }V(t,x(t))\leq -\beta_{3}(\left\|x\right\|),$$ (5)

Where γ∈(0,1), then the equilibrium point of the system (3) is asymptotically stable.

## 3. Stability of time-varying fractional-order systems

### 3.1. Fractional-order system stability analysis

For linear time-varying fractional-order systems, the system (3) can be generally described in the following form:

$${}_{t_{0}}D_{t}^{\alpha }x(t)=A(t)x(t),t\geq t_{0}$$ (6)

For the system (6), we present stability Theorem 3 after introducing Lemma 1 as follows:

**Lemma 1** For a continuous function *f*(*x*) = *x*^*T*^ *Ax*, *x* ∈ *R*^nx1^, if *A*∈ *R*^nxn^ is a positive definite matrix, then

$$\lambda_{\min}(A){\left\|x\right\|}^{2}\leq f(x)\leq \lambda_{\max}(A){\left\|x\right\|}^{2}$$ (7)

in which λ_max_(·) and λ_min_(·) are the maximum and minimum eigenvalues, respectively, of the corresponding matrix.

Theorem 2 provides a guiding stability determination framework for general fractional-order systems, but its complexity is inconvenient when analyzing specific problems. Furthermore, Theorem 1 is not suitable for time-varying systems (6) [21]. Hence, for the time-varying fractional-order system (6), a stability analysis method will be given, we define a real symmetric matrix *H(t)*as follows:

$$H(t)=A(t)+A^{T}(t)$$ (8)

As *H(t)* is a real symmetric matrix, whose eigenvalues are real numbers. Let λ_min_ and λ_max_ be the respective minimum and maximum eigenvalues of *H(t)*. We thus obtain the following asymptotic stability sufficiency with Theorem 3.

**Theorem 3** The sufficient condition for asymptotic stability of the fractional-order system (6) with equilibrium point *x*_*e*_=0 is that the maximum eigenvalue of *H(t)* satisfies λ_max_ < 0.

**Proof:** Let $V(x,t)=x^{T}(t)x(t)={\left\|x(t)\right\|}^{2}$,

Taking the *α*-order derivative of V(x,t)with respect to time *t*, we have

$$\begin{array}{l} V^{\alpha }(x,t)=\frac{d^{\alpha }}{dt^{\alpha }}x^{T}(t)\cdot x(t)+x^{T}(t)\cdot \frac{d^{\alpha }}{dt^{\alpha }}x(t)\\ =x^{T}(t)\left[A^{T}(t)+A(t)\right]x(t)\\ =x^{T}(t)H(t)x(t) \end{array}$$

As λ_min_ and λ_max_are the minimum and maximum eigenvalues of the real symmetric matrix ***H(t)***, respectively, according to **Lemma 1**, we have

$$\lambda_{\min}{\left\|x(t)\right\|}^{2}\leq x^{T}(t)H(t)x(t)\leq \lambda_{\max}{\left\|x(t)\right\|}^{2}$$

Therefore $V^{\alpha }(x,t)\leq \lambda_{\max}{\left\|x(t)\right\|}^{2}$

Considering the theorem’s condition λ_max_ < 0, we can easily obtain the conclusion because *V(x*,*t)*satisfies the condition (1) of Theorem 2, and *V*^α^(x,t) satisfies the condition (2) of Theorem 2. Hence, according to Theorem 2, the time-varying fractional-order system (6) with equilibrium point *x*_*e*_ is asymptotically stable.

### 3.2 Examples of fractional-order system stability analysis

The stability analysis of two typical systems is given to demonstrate the effectiveness of the proposed stability theory.

***Example 1***: Consider the linear time-varying fractional-order system (*α* = 0.95)

$${}_{t_{0}}D_{t}^{\alpha }x(t)=\left[\begin{array}{c} ((-b+a)+a\text{cos(}\omega t))x_{\text{1}}+(b-a\text{sin(}\omega t))x_{\text{2}}\\ (-b-a\text{sin(}\omega t))x_{\text{1}}+((-b+a)-a\text{cos(}\omega t))x_{\text{2}} \end{array}\right].$$ (9)

The system matrix of (9) is

$$A(t)=\left[\begin{array}{cc} \left(-b+a)+a\text{cos(}\omega t\right) & b-a\text{sin(}\omega t)\\ -b-a\text{sin(}\omega t) & \left(-b+a)-a\text{cos(}\omega t\right) \end{array}\right].$$

We assume that a = 0.25, b = 1, *ω* = 2, the real symmetric matrix *H(t)* is:

$$H(t)=A(t)+A^{T}(t)=\left[\begin{array}{cc} -1.5+0.5\cos(2t) & -0.5\sin(2t)\\ -0.5\sin(2t) & -1.5-0.5\cos(2t) \end{array}\right].$$

The eigenvalues of *H(t)*can be obtained:

$$\lambda_{1}=-1,\lambda_{2}=-2.$$

All the eigenvalues are negative, we can conclude that system (9) is stable from Theorem 3.

***Example 2***: Consider the following linear time-varying fractional-order system

$${}_{t_{0}}D_{t}^{\alpha }\dot{x}(t)=\left[\begin{array}{c} (-b+a\text{sin}\omega t)x_{\text{1}}+a(\text{cos}\omega t)x_{\text{2}}\\ a(\text{cos}\omega t)x_{\text{1}}+(-b-a\text{sin}\omega t)x_{\text{2}} \end{array}\right].$$ (10)

The system matrix *A(t)* and the corresponding real symmetric matrix *H(t)* of (10) are acquired as follows:

$$A(t)=\left[\begin{array}{cc} -b+a\text{sin}\omega t & a\text{cos}\omega t\\ a\text{cos}\omega t & -b-a\text{sin}\omega t \end{array}\right],H(t)=\left[\begin{array}{cc} -2b+2a\text{sin}\omega t & 2a\text{cos}\omega t\\ 2a\text{cos}\omega t & -2b-2a\text{sin}\omega t \end{array}\right]$$

The eigenvalues of *H*(*t*) can be calculated: λ_1,2_ = -2*b*±2a Taking a = 0.25, b = 0.5 (λ_1_ = -0.5,λ_2_ = -1.5),this system is stable according to Theorem 3. And if we take a = 1, b = 0.5(λ_1_ = 1,λ_2_ = -3), it is unable to determine the stability of this system from Theorem 3.

These examples show that Theorem 3 can discriminate the stability of time-varying fractional-order systems accurately. However, it is worth noting that this theorem is only a sufficient condition rather than a sufficient and necessary condition.

## 4. Control and synchronization of fractional-order Lü chaotic system

With the global boom of complex network research [27], chaotic systems as a part of complex networks are being widely applied [28]. The robust control and synchronization of chaotic systems have been gaining increasing attention. However, because of the lack of a stability analysis method for fractional-order systems, no systematic solution exists for the control and synchronization of a fractional-order chaotic system. With the use of the time-varying fractional-order stability theorem proposed in this paper, two controllers are designed for the fractional-order Lü chaotic system’s tracking control and synchronization.

### 4.1 Tracking control of fractional-order Lü chaotic system

The mathematic model of fractional-order Lü chaotic system is described as follows [26]:

$${}_{t_{0}}D_{t}^{\alpha }x(t)=g(x)=\left[\begin{array}{c} 30(x_{2}-x_{1})\\ -x_{1}x_{3}+22.2x_{2}\\ x_{1}x_{2}-8.8x_{3}/3 \end{array}\right].$$ (11)

Evidently, the above system is a typical nonlinear fractional-order system. To make the fractional-order Lü chaotic system (11) stable, the controller is designed as follows:

u(t)=Kx(t). (12)

Where u = [u_1_,u_2_,u_3_]^T^, x = [x_1_,x_2_,x_3_]^T^, *k* = [*k*_1_,*k*_2_,*k*_3_]^T^, and the real number *k*_1_,*k*_2_,*k*_3_ must be selected properly. By exerting the control action (12) into the system (11), we obtain

$${}_{t_{0}}D_{t}^{\alpha }x(t)=\left[\begin{array}{c} 30(x_{2}-x_{1})+u_{1}\\ -x_{1}x_{3}+22.2x_{2}+u_{2}\\ x_{1}x_{2}-8.8x_{3}/3+u_{3} \end{array}\right].$$ (13)

We then simplify the controller as u_1 =_ 0,u_2_ = kx_2_,u_3_ = 0 Single-variable linear feedback needs to be used to control the system. The controlled fractional-order Lü system can thus be written in the form of the following linear time-varying fractional-order system:

$${}_{t_{0}}D_{t}^{\alpha }x(t)=\left[\begin{array}{ccc} -30 & 30 & 0\\ 0 & k+22.2 & -x_{1}\\ 0 & x_{1} & -8.8/3 \end{array}\right]\left[\begin{array}{c} x_{1}\\ x_{2}\\ x_{3} \end{array}\right].$$ (14)

The matrix ***A(t)*** of the controlled system (14) is

$$A(t)=\left[\begin{array}{ccc} -30 & 30 & 0\\ 0 & k+22.2 & -x_{1}\\ 0 & x_{1} & -8.8/3 \end{array}\right]$$ (15)

The matrix ***A(t)*** the control parameter *k* and is a function of the state variable x_1_. Thus, it is a time-varying matrix even if the control parameter *k* is fixed. According to Eq 8, ***H(t)***is

$$H(t)=\left[\begin{array}{ccc} -60 & 30 & 0\\ 30 & 2(k+22.2) & 0\\ 0 & 0 & -2*8.8/3 \end{array}\right]$$ (16)

By solving *det*(*λI*−*H*(*t*)) = 0, we obtain the eigenvalues of ***H(t)***.Combining the root locus analysis, we determine that all the eigenvalues of ***H(t)*** are less than 0 if *k*<−29.7. According to Theorem 3, the controlled Lü system (13) is uniformly asymptotically stable.

**Fig 1:**
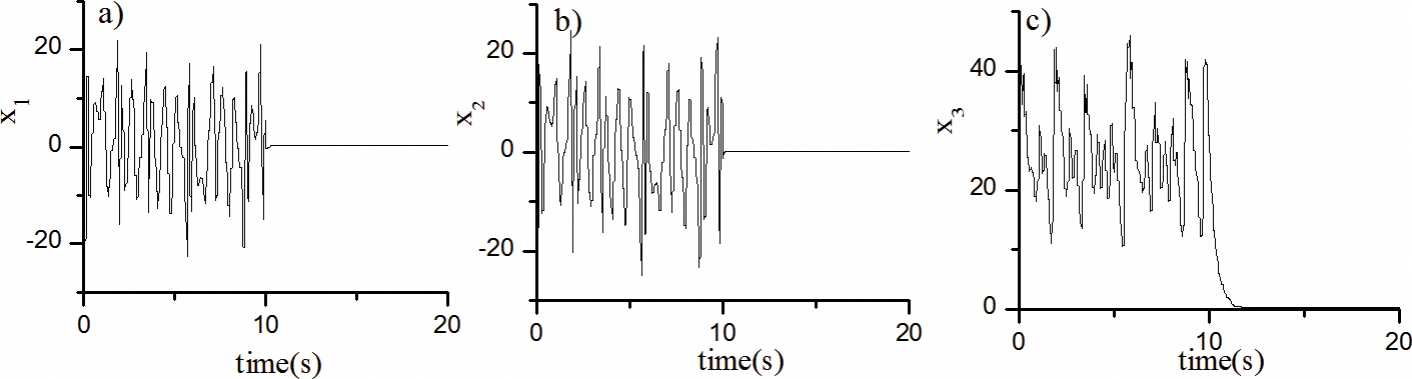
The state diagram of fractional-order Lü chaotic system with robust controller. The motion curves of each state of the fractional-order Lü chaotic system when control action is added at *t* = 10s.

Fig 1. shows the fractional-order Lü system (13) controlled to the zero equilibrium point with *k* = -35.

The solid lines in Fig 1. shows the motion curves of each state of the fractional-order Lü chaotic system when control action is added at *t* = 10s. Clearly, the system gradually converges to equilibrium point S_0_ = (0,0,0) after the control action is added. The above design shows that we can easily obtain a feedback control parameter *k* to make the system stable using ***H(t)***-based Theorem 3. Given the time-varying state *x*_1_ contained in ***A(t)***, obtaining a feedback control parameter *k* using ***A(t)***-based Theorem 1 is difficult.

### 4.2 Synchronization of fractional-order Lü chaotic system

System (11) is selected as the driving system

$${}_{t_{0}}D_{t}^{\alpha }x(t)=\left[\begin{array}{c} 30(x_{2}-x_{1})\\ -x_{1}x_{3}+22.2x_{2}\\ x_{1}x_{2}-8.8x_{3}/3 \end{array}\right].$$ (17)

The response system is

$${}_{t_{0}}D_{t}^{\alpha }y(t)=\left[\begin{array}{c} 30(y_{2}-y_{1})+u_{1}\\ -y_{1}y_{3}+22.2y_{2}+u_{2}\\ y_{1}y_{2}-8.8y_{3}/3+u_{3} \end{array}\right].$$ (18)

The synchronization error is defined as follows:

$$e_{1}=y_{1}-x_{1},\;e_{2}=y_{2}-x_{2},\;e_{3}=y_{3}-x_{3}.$$

Our purpose is to design u(t) = [u_1_,u_2_,u_3_]^T^ to obtain $\underset{t\to \infty }{\lim}\left\|e\right\|=\underset{t\to \infty }{\lim}\left\|y-x\right\|=0$.Then, the error system is

$${}_{t_{0}}D_{t}^{\alpha }e(t)=\left[\begin{array}{c} 30(e_{2}-e_{1})+u_{1}\\ x_{1}x_{3}-y_{1}y_{3}+22.2e_{2}+u_{2}\\ y_{1}y_{2}-x_{1}x_{2}-8.8e_{3}/3+u_{3} \end{array}\right]=\left[\begin{array}{c} 30(e_{2}-e_{1})+u_{1}\\ -y_{3}e_{1}-x_{1}e_{3}+22.2e_{2}+u_{2}\\ y_{2}e_{1}+x_{1}e_{2}-8.8e_{3}/3+u_{3} \end{array}\right].$$ (19)

The controller is designed as u_1_ = 0,u_2_ = y_3_e_1_+ke_2_,u_3_ = -y_2_e_1_.The objective is to use a simple signal feedback control to synchronize the systems. The controlled fractional-order Lü system can be written as

$${}_{t_{0}}D_{t}^{\alpha }e(t)=\left[\begin{array}{ccc} -30 & 30 & 0\\ 0 & k+22.2 & -x_{1}\\ 0 & x_{1} & -8.8/3 \end{array}\right]\left[\begin{array}{c} e_{1}\\ e_{2}\\ e_{3} \end{array}\right].$$ (20)

**Fig 2:**
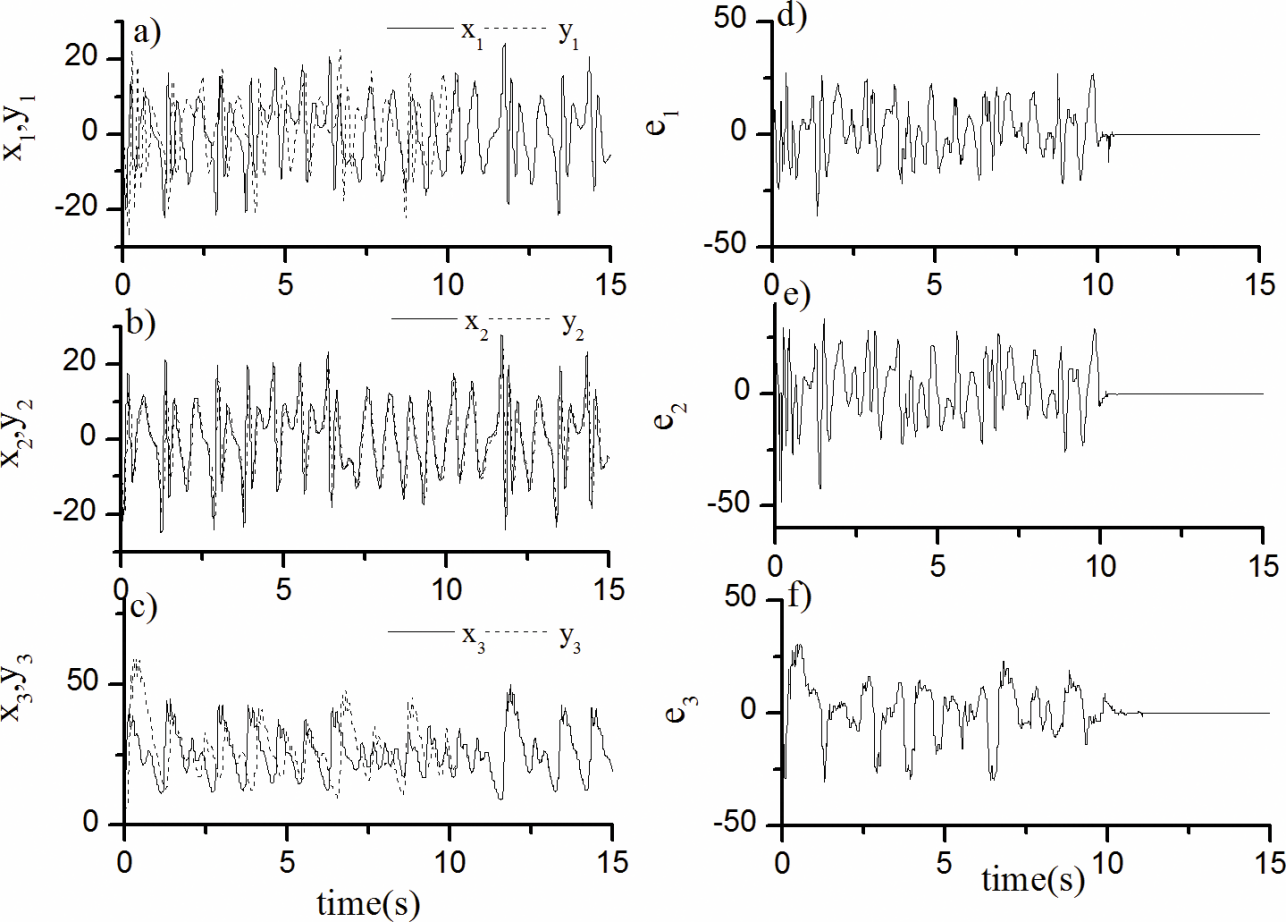
Synchronization results of fractional-order Lü chaotic system. The motion curves of each state of the fractional-order Lü chaotic driving system and response system when the control action is added at *t* = 10 s.

In accordance with the design process of the tracking controller in Section 4.1, the same feedback coefficient *k* can guarantee the stability of the synchronization systems. The synchronization results of the fractional-order Lü system when k = -35 are shown in Fig 2.

The solid lines in Fig 2 show the motion curves of each state of the fractional-order Lü chaotic driving system and response system when the control action is added at *t* = 10 s. Clearly, the response curves tend to the driving curves after the control action is added. The error curves in sub-figures d)–e) of Fig 2 show the quickness and effectiveness of the method.

The above design shows that we can easily obtain a feedback control parameter *k* to make the system stable according to the proposed method. However, the time-varying state *x*_1_ is contained in ***A(t)***, so it is difficult to obtain a feedback control parameter *k* based on Theorem 1.

## 5. Conclusion

A sufficient stability theorem for time-varying fractional-order systems is proposed because the existing stability determination methods for fractional-order systems are complicated and difficult to apply. On the basis of the proposed theorem, a feedback controller for the fractional-order Lü chaotic system is designed for tracking control and synchronization. Simulation results demonstrate the effectiveness of the method.

## Supporting information

S1 Data (ZIP)
